# Financial Data Center Configuration Management System Based on Random Forest Algorithm and Few-Shot Learning

**DOI:** 10.1155/2022/9051629

**Published:** 2022-01-28

**Authors:** Xinxin Li, Lina Wang

**Affiliations:** ^1^Capital University of Economics and Business, Beijing 100070, China; ^2^School of Financial Technology, Hebei Finance University, Baoding, Hebei 071051, China

## Abstract

To form a unified configuration and information management platform, FCCMS (financial center configuration management system) will integrate and sort information based on various configuration data and relationships as well as integrate processes and permissions. However, the most serious issue that data centers are currently facing is how to effectively manage these infrastructures. For various infrastructures, the data center currently uses a decentralized operation and maintenance management model. When an infrastructure fails due to inexperienced configuration management, this mode is not conducive to quickly locating and resolving the problem. A detection method of RFCO (random forest algorithm based on clustering optimization) is proposed, and an appropriate tree is selected from RF to integrate, so as to achieve the best effect. In this paper, the target matching algorithm based on FSL (few-shot learning) is deeply studied, and the target detection model is applied to the target matching and positioning task by using the ML method. The performance of the algorithm is tested by experiments on relevant datasets to verify the effectiveness of the algorithm in various scenarios.

## 1. Introduction

In China, the business volume of major commercial banks has increased significantly year after year as banking business has become more integrated with local market demand and with the global financial environment. At the same time, the banking industry's reliance on IT systems is becoming increasingly apparent [1]. Due to the large number of application systems and servers, as well as the large scale of supporting network rooms, it is often difficult to quickly locate the causes of emergencies, analyze the true root causes of problems, and influence related modules due to poor consideration in upgrading and changing, making it difficult to ensure the high availability and work efficiency of data center systems [2, 3].

Many excellent algorithms have appeared in the field of computer vision, and these algorithms have made remarkable achievements in the fields of image classification, target location, target detection, etc. However, the establishment of many algorithms depends on a large amount of training data, but it is difficult to get rich data in practical application scenarios [4–6]. Usually, the change of financial data reflects this gradual process information; therefore, some algorithms can be used to analyze financial data and design a system that can scientifically reflect the company's financial status, so as to guide enterprise decision makers to formulate correct guidelines, improve business activities, and prevent such problems in time [7]. DL (deep learning) has begun to rise. The related algorithms based on this have found a breakthrough for FSL (few-shot learning) [8, 9]. We establish a data center configuration management system in line with this practice standard and finely manage various infrastructures, so as to establish a long-term mechanism of production and operation with safety production as the core and effectively improve the service level of the data center.

Currently, each department's configuration information is primarily managed by hand. Because there is no unified configuration information maintenance window, there will be unnecessary redundancy when the configuration information is maintained by different departments. Furthermore, the data center's massive IT resources will result in high manual maintenance costs [10]. Although the trading platform system has an automatic monitoring platform, analyzing the impact of events is difficult due to the lack of a systematic relationship between configurations and the corresponding relationship between levels of technical business configuration units. The FCCMS (financial center configuration management system) uses the RF (random forest) algorithm and FSL as the guiding framework to design, classify, and model various infrastructures, abstract them into various configuration items, give them appropriate attributes and names, and establish the relationship between configuration items.

## 2. Related Work

The univariate analysis model takes a single indicator variable, compares financial data from businesses in various stages of crisis, eliminates insignificant financial indicators, and determines the financial indicators with the best discrimination ability [11]. After the analysis, the conclusions are not completely consistent due to the different research samples selected by different researchers. According to the literature [12], the three financial indicator pairs of shareholders' equity/assets and liabilities, working capital/total capital, and current assets/current liabilities have the best ability to predict financial crises. The results of a study [13] comparing and analyzing four financial ratio indicators show that the current ratio and debt ratio have the best early warning effect. The improved fruit fly optimization algorithm is combined with the *Z*-score model to create a financial early warning model in literature [14], and the results show that the model has good forecasting ability. The neural network model is used in literature [15] to study enterprise financial crisis early warning and is compared to the multivariate discriminant analysis model. The results show that the neural network model's prediction effect is clearly greater than that of the multivariate discriminant analysis model. The financial crisis prediction model based on moderate financial indicators and a genetic algorithm is studied in the literature [16], and the results show that this type of model has a high prediction accuracy. Literature [17] compares an SVM-based early warning model to a neural network model and a multivariate discriminant analysis model. The results show that SVM outperforms other models in terms of prediction accuracy. In [18], bagging and boosting methods were used to create a financial crisis early warning model. In [19], the dimension of the index system is reduced through PCA (principal component analysis), and the top three financial indicators in order of importance are turnover per share, asset turnover rate, and current ratio.

In the financial data center, when the number of equipment systems in an IT environment increases to a certain scale, the system management team will definitely consider enabling appropriate tools to assist management. Mihelcic et al. [20] studied an online regression algorithm of nondistributed sampling. Lee and Moon [21] put forward the idea of nonindependent identical distribution in data analysis and processing and other related fields and formed a set of nonindependent identical distribution learning framework. Zhu et al. [22] proposed a corner extraction algorithm, which realized corner detection. Panigrahy et al. [23] obtained a model that can accurately match complex scenes through a large amount of data. By introducing DenseNet, the number of parameters is reduced and the training complexity of target detection network is optimized.

## 3. Research Method

### 3.1. Overall System Design

#### 3.1.1. Basic Architecture Design of the System

**Figure 1:**
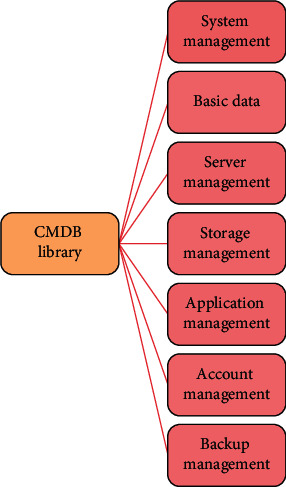
System function module diagram.

All data centers are attempting to reduce operating and maintenance costs, and service integration has proven to be effective in practice. Reduced energy consumption and the implementation of virtualization are two strategies that can effectively reduce the cost of operation and maintenance. The specific implementation of these measures, on the other hand, must be based on a clear understanding of how many servers are running and which servers are deployed, that is, by establishing a system to share information and data, reflecting data relationships, and forming a unified data center with configuration information. System function modules are shown in Figure 1.

In the design of CMDB (configuration management database) configuration items, the query function should be considered, and the additional functions developed according to the outstanding query requirements of customers should be customized, including the following specific functions: query the configuration item list according to the configuration item type and attribute conditions and search the target configuration item list according to the specific relationship between the configuration items. You can filter the attribute conditions in the target configuration item list and import and export the query configuration for any related level configuration item query to facilitate repeated query requirements in the future.

From the perspective of the system administrator, combined with blocky information, users can quickly enter the system and use and complete system functions, such as server administrator, which is concerned with service configuration, which application services are attached to the server, which storage is attached to the server, and how the server is maintained, among other things. The database administrator is concerned with the database's character section, which applications receive database services, which databases each application corresponds to, and which application databases offer cluster services.

#### 3.1.2. Hardware Architecture of the System

The configuration management system of the data center adopts the three-tier architecture of client, server, and NAS storage, in which the client is installed on the PC terminal that the operation and maintenance personnel use daily, and the operation and maintenance personnel of the data center can log in to the configuration management system by double-clicking the client, and the interface is intuitive and friendly and easy to operate.

**Figure 2:**
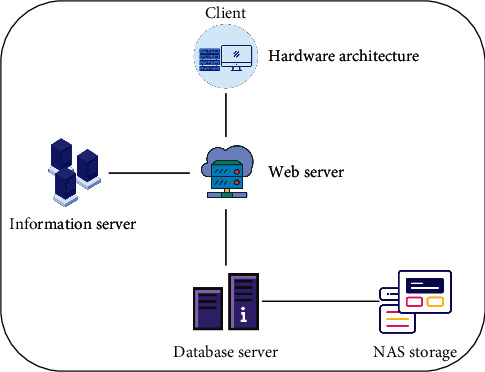
Configuration of the hardware architecture of the management system.

As the core layer of the whole configuration management system, the web server is mainly responsible for responding to various requests submitted by clients, calling database services in the background by triggering events, and then the database server is responsible for data operation. All kinds of data information are finally stored in NAS storage. The hardware architecture of the configuration management system is shown in Figure 2.

The configuration management system uses 8 PC servers with high configuration of 48C96G as web servers, which are responsible for responding to requests from browsers, and 3 databases are installed with SQL Server 2005 database products. Client-server adopts B/S architecture which is widely used at present. The advantage of this architecture is that it is easy to operate and the client needs no maintenance.

#### 3.1.3. System Security Architecture Design

**Figure 3:**
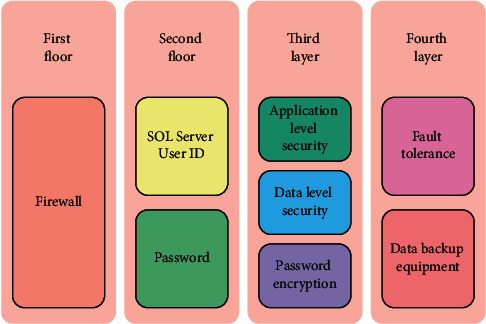
Deployment diagram of system architecture.

Information security is the top priority for system construction. First of all, the most basic security measure is to establish an antivirus mechanism covering the whole network. In addition, a public four-tier security system model is proposed. In this four-layer model, the emphasis of each layer is different, as shown in Figure 3.

The first step of network security protection is planning. It is necessary to divide the trusted network and untrusted network from the business point of view, or the network area can be layered according to the distributed method, and each layer has different protection methods. Firewall devices are used to control or directly isolate users and services in different trust levels of network segments.

If users need access to the data center configuration library application system, they must first go through an identity authentication process. The most basic method is for the user to submit some identity authentication information to the application system in charge of identity authentication, and the application system compares that information to the correct data stored in the system. The user will be allowed to enter the configuration library application system if the verified information is legal and valid; otherwise, the user will be denied.

The creation, modification, deletion, and enabling and disabling of user accounts are all part of user management. Flexible operation and ease of use are required for user management. Grouping and authorizing user accounts, managing and maintaining permissions for user groups or roles, defining and maintaining permissions, and so on are all examples of permission management. Permission management necessitates a high level of security, accessibility, and hierarchical authorization.

### 3.2. Key Algorithm Design

#### 3.2.1. Implementation of System Security Algorithm

The accuracy of member classifiers and the diversity of classifiers in classifier integration system are important issues that researchers have been paying attention to. Similarly, it is difficult to obtain satisfactory classification results by combining some classifiers with different distributions of classification errors but low performance.

RF algorithm is a classifier algorithm based on decision tree. RF has two kinds of important randomicity, the randomicity of training set extraction and the randomicity of node candidate segmentation feature set, which ensure the diversity of decision trees that make up RF. In addition, RF is a data-driven nonparametric classification method, which does not need prior knowledge, only needs to train and classify the given samples, and has a clear structure and is easy to understand.

The main idea of RF algorithm based on clustering optimization is to combine the two base classifiers with the highest correlation degree into a new cluster by using clustering algorithm based on correlation degree and then recalculate the correlation degree between the new clusters. By combining the base classifiers with high correlation, the RF model of aggregation based on correlation measure can be obtained. RFCO (random forest algorithm [25] based on clustering optimization) algorithm is mainly divided into three steps:

1. Firstly, a large number of base classifiers are generated by RF algorithm, and these base classifiers are added to RF to prepare for the next clustering analysis.
2. According to the similarity calculation rules between base classifiers, the similarity of base classifiers is calculated. Then, sort the similarity of all base classifiers to find out the two base classifiers with the greatest similarity.
3. According to the results of clustering analysis, the algorithm will continue to merge the base classifiers, and when the predetermined termination condition is reached, the algorithm clustering will be terminated. Combining all cluster representatives, the RF model after cluster optimization is obtained.

According to the law of large numbers, when *m*⟶*∞*, the function converges.

(1) $$\begin{array}{c} \frac{1}{m}\sum_{i=1}^{m}\alpha_{i}\left(H\left(x;\pi_{i}\right)=j\right),\;j=1,2,\ldots ,J. \end{array}$$

Let the *n*-th base learning model not included in the *m* training sets be *H*^*n*^(*x*;*π*_*i*_), *i*=1,  2,   …,  *m*; then, the strong learning model without included sample combination can be expressed as follows:

(2) $$\begin{array}{c} H_{m}^{n}\left(x\right)=\text{arg}\underset{j}{\text{max}}\frac{1}{M}\sum_{\mathrm{i=1}}^{M}\alpha_{i}I\left(H^{n}\left(x\text{;}\pi_{i}\right)=j\right). \end{array}$$

Therefore, the generalization error of RF algorithm based on clustering optimization can be estimated by test set. The generalization error of RF algorithm based on clustering optimization is constructed by training set as follows:

(3) $$\begin{array}{c} \frac{1}{N}\sum_{n=1}^{N}I\left(H_{m}^{n}\left(x_{n}\right)\neq y_{n}\right). \end{array}$$

Therefore, network intrusion detection based on clustering optimized RF algorithm does not need to divide the collected network intrusion data into training set and test set. Furthermore, the optimized RF algorithm based on clustering and the optimal RF algorithm based on fruit fly are two separate algorithm implementation processes that do not clash, so they can be used together to improve the accuracy of network intrusion detection.

#### 3.2.2. ML-Based Target Matching

With the development of machine learning, deep neural network is widely used. However, because deep neural network is easy to overfit in scenes with small sample size, some techniques to prevent overfitting in scenes with small sample size have been proposed. At present, among FSL methods in the field of computer vision, Bayesian learning, measurement network, ML (machine learning algorithm), and other methods are the mainstream methods.

For FSL problem, ML mainly uses the existing knowledge to solve the learning problem in the scene where there is only a small amount of data in the target field, and there have been many related technical achievements at present. For example, using self-learning technology, using a large number of unlabeled data in the source domain and samples in the target domain, and building an automatic sparse encoder to extract high-level features from samples, the task effect in the target domain is improved. According to the difference between the source domain and the target domain, the model structure is modified and different cost functions are set to get better results in the target domain. At present, the parameter migration method is widely used in image classification, target detection, and other tasks.

In this paper, a method of fast image transformation and solving the transformed target position is proposed. The specific affine matrix is generated according to the following formula:

(4) $$\begin{array}{c} M=M_{\mathrm{scale}}M_{\mathrm{rota}}M_{\mathrm{shear}}M_{\mathrm{trans}}=\left[\begin{array}{ccc} \theta_{11} & \theta_{12} & \theta_{13}\\ \theta_{21} & \theta_{22} & \theta_{23} \end{array}\right], \end{array}$$

where *M*_scale_ is the scale transformation matrix, *M*_rota_ is the rotation matrix, *M*_shear_ is the staggered transformation matrix, and *M*_trans_ is the spatial translation matrix. By simulating the parameters of each matrix and then using the spatial transformation network transformation, the image can be simulated comprehensively by affine transformation.

At the same time, a row of $\left[\begin{array}{ccc} 0 & 0 & 1 \end{array}\right]$ is filled under each spatial transformation matrix *M*, and the corresponding inverse matrix is generated for later calculation of the position of the transformed target, and the inverse matrix form is shown in the following formula:

(5) $$\begin{array}{c} M_{\text{inv}}=\left[\begin{array}{ccc} \theta_{11}^{\prime } & \theta_{12}^{\prime } & \theta_{13}^{\prime }\\ \theta_{21}^{\prime } & \theta_{22}^{\prime } & \theta_{23}^{\prime }\\ \theta_{31}^{\prime } & \theta_{32}^{\prime } & \theta_{33}^{\prime } \end{array}\right]. \end{array}$$

When the input image is transformed by the spatial transformation network according to the matrix *M*, the target position in the new image will also change. The rectangular frame of the original target will become a parallelogram. The new target center position needs to be calculated. The calculation formula is shown in the following formula:

(6) $$\begin{array}{c} \left[\begin{array}{c} x_{gt^{\prime }}\\ y_{gt^{\prime }}\\ z_{gt^{\prime }} \end{array}\right]=\frac{\left(M_{\text{inv}}\left(\begin{array}{c} \left(x_{gt}/y_{gt}\right)\times 2\\ 1 \end{array}\right)+1\right)}{2}, \end{array}$$

where (*x*_*gt*′_, *y*_*gt*′_) is the coordinate of the target center in the new image transformed by the spatial transformation network and *z*_*gt*′_ represents irrelevant data. When calculating, first calculate the vector of diagonal vector after transformation, as shown in the following formula.

(7) $$\begin{array}{c} \left[\begin{array}{c} w_{b1}^{tn}\\ h_{b1}^{tn} \end{array}\right]=\left[\begin{array}{cc} \theta_{11}^{\prime } & \theta_{12}^{\prime }\\ \theta_{21}^{\prime } & \theta_{22}^{\prime } \end{array}\right]\left[\begin{array}{c} w_{gt}\\ -h_{gt} \end{array}\right],\\ \left[\begin{array}{c} w_{b2}^{tn}\\ h_{b2}^{tn} \end{array}\right]=\left[\begin{array}{cc} \theta_{11}^{\prime } & \theta_{12}^{\prime }\\ \theta_{21}^{\prime } & \theta_{22}^{\prime } \end{array}\right]\left[\begin{array}{c} w_{gt}\\ -h_{gt} \end{array}\right], \end{array}$$

where (*w*_*b*1_^*tn*^, *h*_*b*1_^*tn*^), (*w*_*b*2_^*tn*^, *h*_*b*2_^*tn*^) represents the vector of the diagonal vector of the target frame in the original image in the transformed image and (*θ*_11_′, *θ*_12_′, *θ*_21_′, *θ*_22_′) is the corresponding element in the inverse matrix *M*_inv_ of the affine matrix.

## 4. Result Analysis and Discussion

### 4.1. System Test Result Analysis

The configuration management system's main functions are data entry, data retrieval, and report generation. The developers will test the module's functionality after it has been developed. Submit the version control code after you have passed the test. Integrate and compile each branch code to form a complete system after all modules have been developed and tested. After the test is completed, the testers will create relevant reports based on the test cases formulated by the demanding personnel.

**Figure 4:**
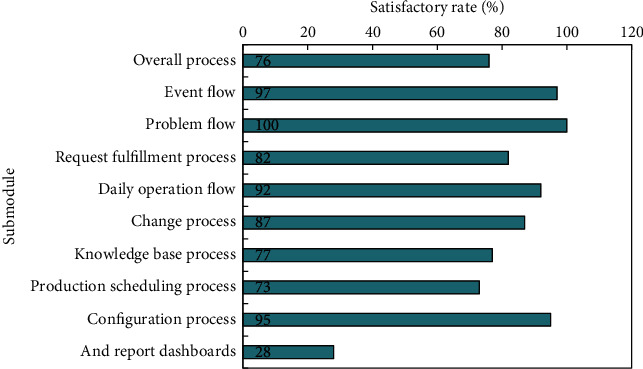
Management system requirements and configuration information integrity analysis table.

The configuration management system of an institution's interbank trading platform has been designed using the configuration management system design scheme. According to the actual test results of the system, the results fully show that the design and implementation scheme of the system can basically meet the requirements of configuration management and can significantly improve the work effect and efficiency in the financial data center, based on the measurement and comparison of the integrity of configuration information and timeliness of collecting configuration data, as well as the representativeness of configuration management requirements in the financial data center. The requirement compliance degree of the configuration management system is shown in Figure 4.

Managing related information of server hardware, configuration, maintenance, and so on is the core module of data center configuration library. All related software programs, services, applications, and so on are inseparable from servers, and the number of servers is often very large. One of the biggest features of the system, such as undeployed related services, supported specific applications, associated storage, change logs, maintenance support, and so on, can be quickly inquired through server links.

**Figure 5:**
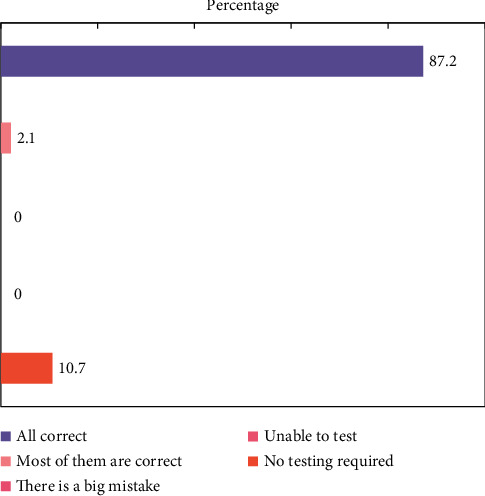
Test result statistics.

After the functional test, integration test, user test, and performance test are completed, and the related problems are solved, and they are finally reflected in the test report. After the test report is signed, it is regarded as an important document of system acceptance. The statistics of test results are shown in Figure 5.

After the configuration management system is put into use, in the quarterly assessment of key performance indicators, such as the variance rate of configuration management database audit, the variance rate of configuration information is always stable at around 1%, which is obviously improved compared with the variance rate of 25%–30% entered manually before.

Secondly, because the configuration management system integrates all kinds of basic information, the operation and maintenance personnel can query related configuration information in time, which saves a lot of precious time for solving incidents and problems and greatly improves the response and solving efficiency of emergencies.

The test results show that the system performs well in terms of scientific and automatic configuration management and IT service management, and the findings of this paper's research should be useful as a practical reference and application for similar data centers with a large number of application systems and infrastructures.

### 4.2. Algorithm Performance Analysis

**Figure 6:**
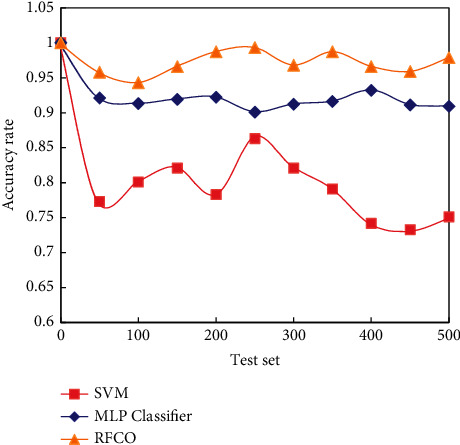
Accuracy comparison of algorithms.

This section mainly describes the intrusion detection capability of RFCO. The experimental results of SVM, MLP classifier algorithm, and RFCO in terms of accuracy are shown in Figure 6.

It can be seen from Figure 6 that the classification accuracy of network intrusion data by RFCO is higher than that of traditional MLP classifier and SVC model.

Storage management is the process of recording storage device configuration information, such as device number, model, supplier, basic configuration, current configuration, maintenance, and other configuration data, as well as the storage's division, use, server connection, and supported business systems. Storage device main table, storage device maintenance information table, storage device original configuration information table, and so on are some of the main designed data tables. Storage device configuration information is kept in the main table, while storage device maintenance information is kept in the storage device maintenance information table.

**Figure 7:**
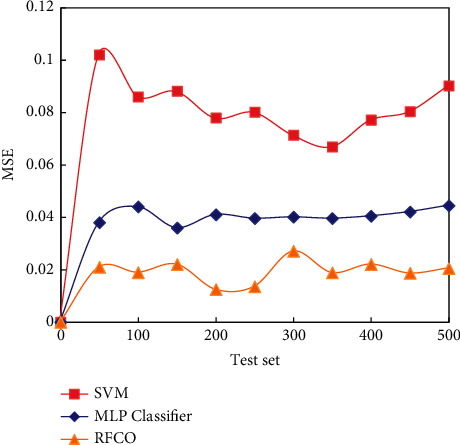
MSE comparison of three algorithms.

Figure 7 compares MSE (mean square error) of three algorithms.

It can be seen from Figure 7 that the MSE value of RFCO for network intrusion detection model is very low. Therefore, the algorithm based on RFCO greatly reduces the MSE of network intrusion detection.

**Figure 8:**
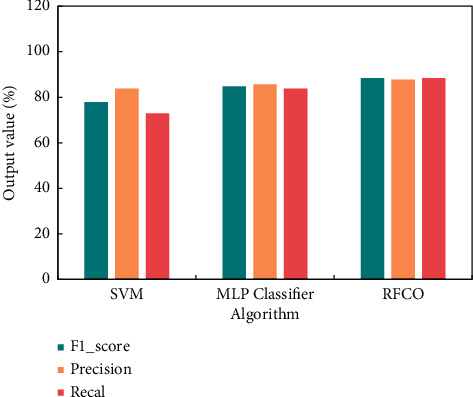
Experimental knot.

These evaluation parameters can reflect the accuracy, error, and various kinds of sample detection of various algorithms to a certain extent, but there are still some shortcomings in measuring the overall performance of the algorithms. Therefore, this section also uses *F*_1__score, precision, and recall parameters to measure the accuracy and robustness of the algorithms in combination with the relevant knowledge of machine learning algorithms. The experimental results are shown in Figure 8.

To sum up, compared with other algorithms, RFCO has a good classification effect, which can effectively solve the problem of various and complex features of network intrusion datasets. For other types of network intrusion data, RFCO also has a good detection effect.

Service management is the process of documenting the related service configuration information that is specifically supported by each application, such as software services, subservices, and the relationship configuration between a service and a specific application, among other things. Software service table, correspondence table between service and application, service relationship table, and so on are the main data tables involved in this module. The software service table primarily stores information about the service type, software name, patch configuration, and service type, among other things. The service and application correspondence table primarily records the relationship between the service and the corresponding application, as well as the service types that it supports, and the service relationship table is linked to related services.

**Figure 9:**
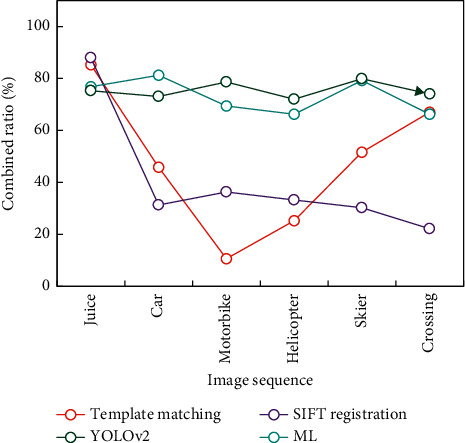
Cross-comparison experiment results.

**Figure 10:**
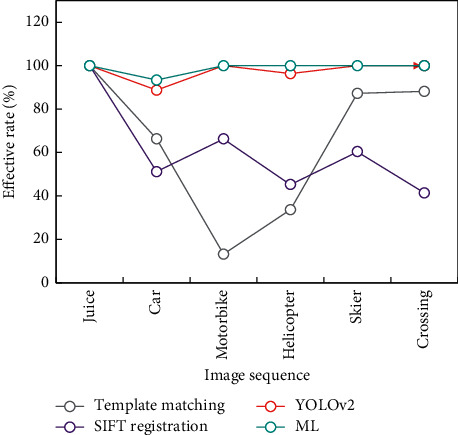
Effective experimental results.

Continue to verify image sequences using the VOT dataset and TempleColor128 dataset, including Juice, Car, and the other six groups. After manually removing the completely occluded and difficult-to-identify images from the above image sequences, about 10 images were chosen as training data and the remaining images were used as test data in the experiment. The intersection ratio results and the algorithm's efficiency results on six groups of image sequences are shown in Figures 9 and 10, respectively.

According to the above experimental data, it can be seen that the traditional algorithm has good effect only in some scenes, while the method based on DL has good generalization ability in all scenes.

In the daily operation of the data center, a series of changes, such as equipment replacement, system migration, project transformation, and so on, are often needed, and all these changes will cause the change of configuration information without exception, so it is necessary to maintain the configuration information to ensure that the currently recorded configuration information is consistent with the actual information in the production environment. Only when the configuration information is accurate can the normal operation of many processes such as service desk, event management, problem management, change management, and release management be supported.

If an existing configuration item template needs to be updated, the process loops back to the configuration item owner, who redesigns the template. The configuration management database administrator updates the configuration item template once the configuration process manager has approved it. The configuration information administrator is authorized to prepare relevant information in accordance with the modified new configuration item template in order to maintain the configuration item after the new configuration item template is established. The old value before the change and the new value after the change should be recorded in the maintenance record table. The name of the configuration item and all other configuration item names associated with the configuration item should be stored in the maintenance record table for a configuration item with the maintenance type “Delete.”

The ML algorithm proposed in this paper maintains a high level of intersection ratio and efficiency. The ML-based target matching algorithm proposed in this paper keeps high efficiency in six scenes. At the same time, it is slightly worse than the previous twin network algorithm in the Car image sequence. The reason for analyzing similar scenes is that the target image and the target image with background are partially used in training, which affects the prediction results when the background changes drastically.

## 5. Conclusion

With the flourishing development of various businesses in the financial industry, particularly with the increasingly active transactions in the financial market and the increasing demand for regulatory statistics, the FCCMS design scheme in this paper has added new explorations and attempts, the theoretical basis and framework of the technical system have become clearer and more systematic, and the support for regulatory statistics has increased. Simultaneously, a large number of highly automated management software programs were chosen, enhancing IT management capability and level. RFCO synthesizes and optimizes the model's base classifiers, improving network intrusion detection accuracy. Finally, the target matching algorithm based on ML network proposed in this paper has significantly improved efficiency over traditional methods and has higher matching and positioning accuracy, according to the experimental results. The performance of small targets and scenes with spatial transformation has also been greatly improved when compared to the original YOLOv2 algorithm.

The FCCMS is currently unable to achieve full automation, and maintenance tasks such as establishing configuration item correlations and changing configuration item status and attributes still require manual intervention. We will strive to integrate the knowledge base, problem report, and other functions into the configuration management system in the future exploration so that the configuration management system can be more closely integrated with other processes and the data center's IT service quality can be improved further.

## Data Availability

The data used to support the findings of this study are included within the article.

## References

[1] Faiz T. I., Noor-E-Alam M. (2019). Data center supply chain configuration design: a two-stage decision approach. *Socio-Economic Planning Sciences*.

[2] Cheung H., Wang S. (2019). Optimal design of data center cooling systems concerning multi-chiller system configuration and component selection for energy-efficient operation and maximized free-cooling. *Renewable Energy*.

[3] Garnica G. (2018). Configuration management. *Naval Engineers Journal*.

[4] Ambroise L., Prim-Allaz I., Teyssier C. (2018). Financial performance of servitized manufacturing firms: a configuration issue between servitization strategies and customer-oriented organizational design. *Industrial Marketing Management*.

[5] Guo H., Gao S., Tsui K. L. (2017). Simulation optimization for medical staff configuration at emergency department in Hong Kong. *IEEE Transactions on Automation Science and Engineering*.

[6] Dai B., Xu G., Huang B., Qin P., Xu Y. (2017). Enabling network innovation in data center networks with software defined networking: a survey. *Journal of Network and Computer Applications*.

[7] Li X. Y., Liu Y., Lin Y. H. (2020). A generalized petri net-based modeling framework for service reliability evaluation and management of cloud data centers. *Reliability Engineering & System Safety*.

[8] Huang C., Huang Q., Wang D. (2019). Stochastic configuration networks based adaptive storage replica management for power big data processing. *IEEE Transactions on Industrial Informatics*.

[9] Nadjahi C., Louahlia H., Lemasson S. (2018). A review of thermal management and innovative cooling strategies for data center. *Sustainable Computing: Informatics and Systems*.

[10] Trestian R., Katrinis K., Muntean G. M. (2017). OFLoad: an OpenFlow-based dynamic load balancing strategy for datacenter networks. *IEEE Transactions on Network & Service Management*.

[11] Cheng Y., Qiao X., Wang X. (2017). Random forest classifier for zero-shot learning based on relative attribute. *IEEE Transactions on Neural Networks and Learning Systems*.

[12] Xu H., Wang J., Li H., Ouyang D., Shao J. (2021). Unsupervised meta-learning for few-shot learning. *Pattern Recognition*.

[13] Trivedi A., Bovornkeeratiroj P., Breda J., Shenoy P., Taneja J., Irwin D. (2021). Phone-based ambient temperature sensing using opportunistic crowdsensing and machine learning. *Sustainable Computing: Informatics and Systems*.

[14] Feng R., Zheng X., Gao T. (2021). Interactive few-shot learning: Limited supervision, better medical image segmentation. *IEEE Transactions on Medical Imaging*.

[15] Sun-yuan Q., Da-jun L., Chen K. C., Zeng C. (2019). QR code reconstruction algorithm based on binary random forest under non-uniform illumination. *Packaging Engineering*.

[16] Park J., Yi S., Choi Y., Cho D. Y., Kim J. (2019). Discriminative few-shot learning based on directional statistics. http://arxiv.org/abs/1906.01819.

[17] Li X., Rao Y., Wang W., Feng C. (2020). SLBCNN: a improved deep learning model for few-shot charge prediction. *Procedia Computer Science*.

[18] Paul A., Mukherjee D. P., Das P., Gangopadhyay A., Chintha A. R., Kundu S. (2018). Improved random forest for classification. *IEEE Transactions on Image Processing*.

[19] Lujan-Moreno G. A., Howard P. R., Rojas O. G., Montgomery D. C. (2018). Design of experiments and response surface methodology to tune machine learning hyperparameters, with a random forest case-study. *Expert Systems with Applications*.

[20] Mihelcic M., Dzeroski S., Lavrac N. (2018). Redescription mining augmented with random forest of multi-target predictive clustering trees. *Journal of Intelligent Information Systems*.

[21] Lee S., Moon N. (2018). Location recognition system using random forest. *Journal of Ambient Intelligence and Humanized Computing*.

[22] Zhu C., Cui J., Zou B. (2017). Retinal vessel segmentation based on multiple feature fusion and random forest. *Journal of Computer-Aided Design & Computer Graphics*.

[23] Panigrahy P. S., Santra D., Chattopadhyay P. (2020). Decent fault classification of VFD fed induction motor using random forest algorithm. *Artificial Intelligence for Engineering Design Analysis and Manufacturing*.

[24] Wang Y., Xia H., Yuan X., Li L., Sun B. (2018). Distributed defect recognition on steel surfaces using an improved random forest algorithm with optimal multi-feature-set fusion. *Multimedia Tools and Applications*.

[25] Li D., Zhang P., Zhang Y., Xiao T. (2021). Application of random forest in the analysis of students’ physical health test data. *ASP Transactions on Computers*.

